# Editorial: Atrial Fibrillation: Technologies for Investigation, Monitoring and Treatment, Volume II

**DOI:** 10.3389/fphys.2023.1209458

**Published:** 2023-05-04

**Authors:** Omer Berenfeld, Valentina Corino, Axel Loewe, Juan Pablo Martínez, Jose F. Rodriguez Matas

**Affiliations:** ^1^ Center for Arrhythmia Research, University of Michigan, Ann Arbor, MI, United States; ^2^ Biosignals, Bioimaging and Bioinformatics Laboratory (B3Lab), Department of Electronics, Information and Bioengineering (DEIB), Politecnico di Milano, Milano, Italy; ^3^ Cardio Tech-Lab, Centro Cardiologico Monzino IRCCS, Milan, Italy; ^4^ Institute of Biomedical Engineering, Karlsruhe Institute of Technology (KIT), Karlsruhe, Germany; ^5^ Biomedical Signal Interpretation and Computational Simulation Group, Aragón Institute of Engineering Research, IIS Aragón, Universidad de Zaragoza, Zaragoza, Spain; ^6^ Centro de Investigación Biomédica en Red en Bioingeniería, Zaragoza, Spain; ^7^ LaBS, Department of Chemistry, Material and Chemical Engineering “Giulio Natta”, Politecnico di Milano, Milano, Italy

**Keywords:** atrial fibrillation, mapping, detection, ablation, rate control, prediction, machine learning

## Introduction

Following the great interest in the first edition of the Frontiers Research Topic devoted to technology related to the understanding, management, and therapy of atrial fibrillation (AF), we are glad to present a second collection of additional articles on the Research Topic. AF remains the most common sustained clinical arrhythmia (Chugh et al., 2014; Zoni-Berisso et al., 2014), the leading cause of embolic stroke, and an increased risk for heart failure and mortality (Benjamin et al., 2017; Roth et al., 2017). AF is a major societal and financial burden (Kowalski et al., 2022) likely due to its heterogenous, multifactorial, and progressive nature as well as its suboptimal therapy (Heijman et al., 2018). It has been recently demonstrated that early AF rhythm-control therapies of ablation or antiarrhythmic drugs within the first year of AF diagnosis are beneficial (Eckardt et al., 2022; Dickow et al., 2023) but come at a heavy financial cost (Gottschalk et al., 2023) that is calling for further research and developments. This second volume of the Research Topic continues to aim at the challenge of the burden of AF. It consists of 15 original papers that focus, as with the first edition, on technological advances for the improved understanding of AF and better diagnosis, management, and therapy of arrhythmia. A total of 95 students and faculty of diverse scientific backgrounds have contributed to the papers and highlighted the importance of multidisciplinary research for advancing the understanding and therapy of AF. We are grateful to all the contributors to the first and present Research Topic articles for their time and efforts. The articles in this second volume of the Research Topic can be grouped and are briefly summarized as follows.

## Mapping, characterizing, and predicting atrial fibrillation

It has been proposed that high dominant frequency and rotor regions play a significant role in perpetuating AF. Ehnesh et al. analyzed non-contact recordings from inside the left atrium of 10 persistent AF patients and found that spatial correlation between these two features at any given time is poor but they colocalize in certain regions over time, mainly in the septum. In patients in whom high-dominant frequency ablation terminated the AF, there was some overlapping with longer-lasting rotor sites, but the relationship was complex overall. The study by Jenkins et al. focused on the accuracy of quantifying the lifetime of phase singularity points representing AF re-entrant patterns. The paper argues that the duration of the observation can affect the likelihood of detecting long vs. short lifetimes and suggests that to minimize the dependency on the observation duration, the individual lifetimes, rather than averages of probability distributions, should be considered.

Regions characterized by low voltage activity in atrial mapping are considered to be arrhythmogenic and a target for ablation. Van Schie et al. used a specialized multiple-electrode system to perform intraoperative simultaneous unipolar and omnipolar voltage mapping in opposing endocardial and epicardial right atrial surfaces during sinus rhythm. The study found that the low voltage regions frequently appeared exclusively on either one of the surfaces and suggests that the addition of the omnipolar mapping, where far field and directionality effects are minimized, to the unipolar mapping adds fidelity to the localization of low voltage regions.

The non-invasive electrocardiographic imaging (ECGi) approach is proposed for an effective mapping of AF and ablation guidance but is still a challenging technology. Fambuena-Santos et al. used ECGi and a new rotor detection algorithm to map AF in patients that underwent pulmonary vein isolation. They found that the total rotor number was higher in patients with follow-up arrhythmia recurrence than in those that remained in sinus rhythm and in whom the phase singularity point concentration was higher in the pulmonary vein region. The consistency between the findings on re-entrant pattern distribution and the expected mechanisms suggested the technology could offer useful information.

Efforts to optimize the inverse problem solution at the center of the ECGi approach by various time-frequency decomposition techniques are presented in the article by Yadan et al. the authors applied different mode decomposition algorithms on five simulated and two AF patients datasets to rank the accuracy and efficiency of their performance to hopefully improve the solution of the inverse problem in the clinic.

Approximately 30% of patients undergoing cardiac surgery develop AF post-operatively and predicting who will develop arrhythmia on an individual basis could guide therapeutic management. Kang He et al. developed a machine learning method for the single-lead monitoring of patients after cardiac surgery and concluded that their method could improve the detection of postoperative AF in those patients. Ah Ran Oh et al. also used a machine learning method in post-operative patients, however with non-cardiac surgery, and found that including the top five variables of age, lung operation, operation duration, history of coronary artery disease, and hypertension results in the area under the receiver operating characteristic curve for postoperative AF of 0.80 that indicates the ability of prediction.

Body surface potential maps were processed by McCann et al. to predict catheter ablation outcomes in persistent AF patients. The investigators developed new indices based in part on a principal component analysis to quantify the reconstruction of the full set of the body surface signals with a subset of the signals, as well as a non-dipolar component index. They then associated the levels of those indices with restoring sinus rhythm or recurrence of arrhythmia following single-procedure catheter ablation.

## Atrial fibrillation ablation and drug effects

Radiofrequency (RF) catheter ablation to terminate AF is challenging in some, particularly persistent, AF patients, and efforts are ongoing to increase its success rate. Successful catheter-based RF ablation depends on adequate lesion formation. Alken et al. analyzed contact force and local impedance in 730 RF applications of a novel open-irrigated single-tip catheter in 20 repeat AF ablation patients. A drop of more than 20 Ω in local impedance following the ablation was considered as an adequate lesion formation and was found to be better predictable by the combined rather than a separated force and baseline impedance quantification.

Machine learning methods have been proposed to improve the guidance of ablation. Ogbomo-Harmitt et al. studied the feature attribution of AF data subjected to deep learning algorithms to interpret the results of the algorithms and enhance confidence in the proposed ablation strategy. They used simplified computer models based on patients’ MRI data to suggest that the regions with the most informative features colocalized with pro-arrhythmogenic regions.

In silico studies are also used to interpret and guide pharmacological intervention to terminate AF. Dasi et al. performed simulations on a population of human structurally normal atrial computer models that suggest that AF depends on the formation of discordant alternans. Cardioversion using 10 antiarrhythmic drugs applied to those models was found to be optimal when refractoriness was maximized and was predictable with 70% accuracy from either ionic or ECG properties. Machine learning methods are also used in computer simulation studies to better understand the relationships between pharmacological treatments and the characteristics of the underlying atrial tissue. Sanchez de la Nava et al. used artificial intelligence in a computational proof-of-concept study with the ability to predict susceptibility for conduction blocks in fibrotic atrial tissue and antiarrhythmic drug regime. Simulations on a population of simplified 2D atrial models suggest that fibrosis can alter the expected behavior of several commonly used drugs and that the model profile leading to the different effects might be predicted by data analysis using artificial intelligence.

In most patients, in addition to efforts to terminate AF, concomitant pharmacological efforts are also made to decrease the ventricular rate (Heijman et al., 2021) and would benefit from a better understanding. Karlsson et al. studied the influence of several β-blockers and calcium channel blockers on the atrio-ventricular node conduction in a cohort of permanent AF patients and found differential delays that could be important in treatment selection. On a related topic, Plappert et al. demonstrated that the incorporation of the effects of the autonomic nervous system on conduction in a mathematical model of the atrio-ventricular node is necessary to replicate ventricular rate changes in clinical tilt test data. Abdollahpur et al. investigated respiratory modulation as a surrogate for the autonomic nervous system modulation of the atrial activity during AF by analysis of the f-wave frequency. Their proposed orthogonal subspace projection approach (Varon et al., 2019) showed robustness in detecting respiratory-induced changes on simulated data but results on clinical data were heterogeneous without significant differences between baseline and deep breathing.

## Conclusion

We are excited to present a second Research Topic collection of technology-inclined articles describing recent experimental, computational, and clinical studies on AF. The articles ultimately apply to ablation and pharmacological therapies to restore sinus rhythm or control of the ventricular rate and highlight the need for improved comprehensive management of AF and its associated symptoms (Kirchhof, 2017). We hope that scientists, engineers, and clinicians, as well as patients, interested in the topic, will find this overview of basic and translational research trends to be inspiring and promoting a better understanding of the diagnosis and therapies of AF.
